# An Exported Heat Shock Protein 40 Associates with Pathogenesis-Related Knobs in *Plasmodium falciparum* Infected Erythrocytes

**DOI:** 10.1371/journal.pone.0044605

**Published:** 2012-09-07

**Authors:** Pragyan Acharya, Shweta Chaubey, Manish Grover, Utpal Tatu

**Affiliations:** Department of Biochemistry, Indian Institute of Science, Bangalore, Karnataka, India; Institut national de la santé et de la recherche médicale - Institut Cochin, France

## Abstract

Cell surface structures termed knobs are one of the most important pathogenesis related protein complexes deployed by the malaria parasite *Plasmodium falciparum* at the surface of the infected erythrocyte. Despite their relevance to the disease, their structure, mechanisms of traffic and their process of assembly remain poorly understood. In this study, we have explored the possible role of a parasite-encoded Hsp40 class of chaperone, namely PFB0090c/PF3D7_0201800 (KAHsp40) in protein trafficking in the infected erythrocyte. We found the gene coding for PF3D7_0201800 to be located in a chromosomal cluster together with knob components KAHRP and PfEMP3. Like the knob components, KAHsp40 too showed the presence of PEXEL motif required for transport to the erythrocyte compartment. Indeed, sub-cellular fractionation and immunofluorescence analysis (IFA) showed KAHsp40 to be exported in the erythrocyte cytoplasm in a stage dependent manner localizing as punctuate spots in the erythrocyte periphery, distinctly from Maurer’s cleft, in structures which could be the reminiscent of knobs. Double IFA analysis revealed co-localization of PF3D7_0201800 with the markers of knobs (KAHRP, PfEMP1 and PfEMP3) and components of the PEXEL translocon (Hsp101, PTEX150). KAHsp40 was also found to be in a complex with KAHRP, PfEMP3 and Hsp101 as confirmed by co-immunoprecipitation assay. Our results suggest potential involvement of a parasite encoded Hsp40 in chaperoning knob assembly in the erythrocyte compartment.

## Introduction

The malaria parasite *Plasmodium falciparum* infects human erythrocytes, which are terminally differentiated cells devoid of any organelles. Yet, protein trafficking is important for malaria pathogenesis. The parasite is known to deploy pathogenesis related proteins to the erythrocyte surface [1], [2]. To facilitate protein export, *P. falciparum* establishes its own endomembrane system comprising of ER, Golgi within the parasite and the Maurer’s clefts, tubulovesicular network in the erythrocyte cytosol [3], [4]. The best studied proteins deployed on to the parasite surface are the members of the *var* gene family of proteins that encode for *Plasmodium falciparum* erythrocyte membrane protein 1 (PfEMP1) which mediate cytoadherence of parasitized erythrocytes to uninfected erythrocytes and endothelial cells of the blood vessel [5]. Several molecules of PfEMP1 collect on the infected erythrocyte plasma membrane and closely associate with KAHRP, PfEMP3, host spectrin and actin to form cytoadherent “knobs” [6]. The variable extracellular domains of PfEMP1 interact with host cell receptors such as CD36, ICAM-1 and Chondroitin Sulfate A [reviewed in 7]. Interestingly, the parasite encoded knob components KAHRP and PfEMP3, have unusual amino acid sequences. They contain homorepeats consisting of a specific amino acid within a short peptide stretch, or the presence of Asn/Gln rich prion-like domains, KAHRP having a His-rich N-terminal domain and PfEMP3 consisting of a prion like domain [8]. Such unusual amino acid compositions may predispose these proteins to misfold and aggregate thereby requiring chaperones to stabilize their conformations.

Many exported proteins including knob components contain a penta-peptide export signal known as the PEXEL motif [1], [2]. Recent advances reveal that PEXEL-proteins are transported across the parasitophorous vacuolar membrane (PVM) through an integral membrane PEXEL translocon [9]. The trafficking of PEXEL containing proteins across this translocon may likely involve protein unfolding and refolding events, thereby requiring involvement of chaperones [10]. There is very little known about the export of knob components and mechanism of their assembly. Trafficking of proteins across membranes typically involves the action of several molecular chaperones, generally of the Hsp40 class [reviewed in 11]. However, specific players in the trafficking of knob components have not been studied.

While analyzing the chaperone coding genes of the parasite, we found that of the 18 PEXEL containing Hsp40s, PF3D7_0201800 was present in a cluster with PfEMP3 and KAHRP on the sub-telomeric end of chromosome 2. Chromosomal clusters in the parasite are defined on the basis of identical transcription profile within the same developmental stage of the parasite [12]. Therefore, we decided to explore the role of PF3D7_0201800. Our study revealed that PF3D7_0201800 is a type II J-domain containing protein which is exported in the trophozoite stages of the parasite. Further, we found that the chromosomal clustering of PF3D7_0201800 with PfEMP3 and KAHRP has a functional relevance since this Hsp40 was found to co-localize with KAHRP and PfEMP3 in indirect immunofluorescence analysis (IFA). PF3D7_0201800**,** KAHRP and PfEMP3 were also found to be in a common complex as they co-precipitated in the immunoprecipitation assay. Additionally, we also found that PF3D7_0201800 co-localized with PfEMP1, in structures resembling knobs on the infected erythrocyte membrane.

In all, our study provides first ever biochemical and cell biological evidence for the association of a parasite encoded Hsp40, PFB0090c/PF3D7_0201800 (KAHsp40– Knob Associated Hsp40 in remaining text), with components of knobs in the infected erythrocyte. Our studies implicate the exported Hsp40 to participate in aspects of knob traffic and/or assembly processes.

## Materials and Methods

### Strains and Antibodies

For all experiments, 3D7 strain of *Plasmodium falciparum* was used. *P. falciparum* cultures were maintained as described in [13]. α-KAHsp40 rabbit polyclonal antisera was raised against the C-terminal peptide “IIFPKKLSDEQKELIKEAL” from KAHsp40 protein sequence. α-PFE antibody was raised in rabbit against the C-terminal peptide “KLSPEQKRTLKETLENTY” from the protein sequence of PFE0055c/PF3D7_0501100.1. α-Hsp101 antiserum was raised in mice against the C-terminal peptide “DVFVDYNSKAKNLVINLSKT” from the Hsp101 sequence. α-PTEX150 antiserum was raised in mice against the C-terminal peptide “ENDDDEKGNNNDDENDND” from the PTEX150 sequence. The blots showing the specificity of α-Hsp101 and α-PTEX150 antibodies are given in supplementary data (Figure S1). α-MSP1 antibody (anti-P30P2-PfMSP1-19(Q-KNG) FVO-2 rabbit anti-serum) was obtained from MR4. α-host Hsp70 antibody (SPA810) was obtained from Stressgen. α-KAHRP (mAb 89) antibody, PfEMP1 (α-ATS) antibody, α-MAHRP1 antibody and α-PfEMP3 antibody were kind gifts from Prof. D.Taylor, Prof. Amit Sharma, Prof. Hans Peter Beck and Prof. Alan F. Cowman respectively. The dilutions used for western blotting were 1∶1000 for all primary antibodies and 1∶5000 for secondary antibodies (HRP conjugated – IgG – Rabbit/Mouse: Genei). For IFA, all primary antibodies were used in 1∶50 dilution and secondary antibodies were used in 1∶300 for FITC conjugated and 1∶100 for TRITC conjugated antibody. KAHsp40 IP and KAHRP IP were performed in a dilution of 1∶10 and 1∶100 respectively.

### Protein Purification of KAHsp40

The synthetic gene for KAHsp40 (PEXEL processed form) cloned in the vector pMK-RQ was obtained from Prof. Susan Lindquist. It was sub-cloned in pRSET A vector between Bam H1 and Sac1 restriction sites using the primers 5′-CCCCCCGGATCCATGTCTGCTCAAACTCAAAG - 3′ (forward) and 5′ – CCCCCCGAGCTCTCATTAGAAACCGTTACCTC –3′ (reverse). The His-tagged KAHsp40 construct was transformed into *E. coli* Rosetta strain. The protein was over expressed by induction with 0.1 mM IPTG for 4 h at 37°C. The culture was lyzed by sonication in 50 mM Tris-HCl pH 7.4, 500 mM NaCl, 10% glycerol and 2 mM PMSF with appropriate protease inhibitors. The protein was purified using nickel-nitriloacetic acid (Ni-NTA) affinity chromatography (Qaigen).

### Cell Lysis Protocol

*P. falciparum* cells were lysed with saponin. Saponin is a detergent that allows the selective lysis of the erythrocyte plasma membrane along with PVM and leaves the parasite plasma membrane intact, thereby enabling the separation of erythrocyte cytosol from parasite compartment. In all the experiments conducted here, the infected erythrocytes were separated from the uninfected cells using 60% Percoll gradient.

### Metabolic Labeling and Immunoprecipitations

*P. falciparum* in culture was labeled metabolically with [^35^S]cysteine and -methionine at 200 µCi/ml (BRIT) culture for 16 h. Pulse labeling was carried out by first enriching mature stages on a 60% percoll gradient and then labeling them in [^35^S]cysteine and methionine –free medium (Sigma) for 15 minutes by addition of 500 µCi label. Cells were lysed by saponin and the saponin lysate (SL) and saponin pellet (SP) were separated by centrifugation. The SP was lysed with either denaturing buffer or non-denaturing buffer. The denatured cell lysate was diluted with PBS so that the final concentration of SDS was 0.1% and this was used for immunoprecipitations (IPs). Antibodies were added in appropriate dilutions and incubated at 4°C for 12 h on an end-to-end rotator. Protein A beads (Genei) were then added and incubated for 2 h, at the end of which the beads were washed five times in IP wash buffer (1% Triton X –100 in PBS). After the washes, the immunoprecipitates were eluted by boiling in Laemmli buffer and analyzed by SDS-PAGE. The gels were vacuum dried and exposed in a phosphoimager cassette. The film was scanned after 48 h using a phosphoimager.

### Indirect Immunofluorescence Analysis

Parasite culture at 5–7% parasitemia was smeared on clean glass cover slips and fixed with acetone chilled at −20°C. The smears were blocked with 1% BSA in PBS for 30 min. Primary antibody in PBS was added onto the cover slips and incubated for 90 min. Three washes were given to the cover slips maintaining gentle agitation. Cover slips were then incubated in either FITC-conjugated anti-rabbit secondary antibody alone or along with TRITC-conjugated anti-mouse antibodies for 45 min as required. Three washes were given to the cover slips maintaining gentle agitation. The cover slips were mounted on glass slides with 90% glycerol containing 2% DABCO (and 5 µg/ml propidium iodide wherever required) and visualized under a confocal laser scanning microscope (Zeiss LSM Meta).

### Brefeldin-A (BFA) Treatment

Cultures of *P. falciparum* were synchronized by two consecutive sorbitol treatments, 4 h apart. The cultures were allowed to grow for 12 h post synchronization. BFA dissolved in absolute ethanol was used at a final concentration of 10 µg/ml. Another batch of cells was treated with equal volume of absolute ethanol. The cells were allowed to grow in the presence of BFA for 12 h after which they were smeared on the cover slips for carrying out IFA procedures as described above.

## Results

### KAHsp40 is Present in a Chromosomal Cluster with PfEMP3 and KAHRP

**Figure 1:**
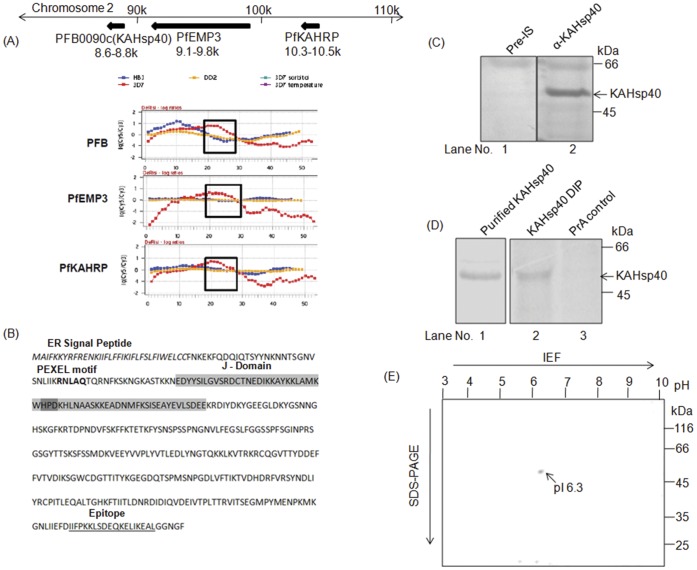
Characteristic features of KAHsp40. (A) Upper panel indicates sub-telomeric chromosomal cluster on chromosome 2 of *Plasmodium falciparum* which consists of KAHsp40, KAHRP and PfEMP3. Lower panel shows the transcription profiles of these genes from different strains of *Plasmodium falciparum* (Adapted from PlasmoDB.org) (B) KAHsp40 sequence consists of an N-terminal ER signal peptide, a PEXEL motif followed by a J-domain that contains a conserved HPD motif. The C-terminal epitope that was used for generating antibody has been underlined. (C) Immunoblot with α-KAHsp40 recognizes a specific band of MW at about 48 kDa in total parasite lysate whereas the pre-immune serum (Pre-IS) does not. (D) The form of KAHsp40 immunoprecipitated from radiolabeled parasite lysate corresponds to the processed form of the protein as its mobility is same as the recombinant protein on SDS-PAGE. Lane 1 is the coomassie stained purified KAHsp40 protein whereas Lanes 2 and 3 is an autoradiogram of IP with α-KAHSp40 antisera and protein-A (PrA) control respectively. (E) Two-dimensional gel of KAHsp40 immunoprecipitated from denatured metabolically labeled infected RBC lysate. DIP- Denaturing Immunoprecipitation.

The gene encoding for KAHsp40 is located on the sub-telomeric end of chromosome 2 in tandem with the genes encoding for PfEMP3 and KAHRP (Fig. 1A upper panel). KAHsp40, PfEMP3 and KAHRP have been previously reported as a co-expressed cluster in the parasite, on the basis of transcriptome analysis (Fig. 1A lower panel, adapted from PlasmoDB.org) [12]. PfEMP3 and KAHRP are integral knob components and are also important for trafficking of PfEMP1 to knobs [14], [15]. KAHsp40 is a Type II Hsp40 that contains an ER signal peptide at the N-terminus, a PEXEL motif (RNLAQ) downstream of it followed by a J-domain (Fig. 1B). The J-domain of KAHsp40 contains all the hallmarks of a typical J-domain including the signature tri peptide HPD which is essential for interaction with an Hsp70 partner (reviewed in 11). The C-terminal peptide from the primary sequence of KAHsp40, marked in Fig. 1B, was used for raising polyclonal antiserum against the protein. As shown in Fig. 1C, the antiserum reacted with a single band having a molecular weight of 48 kDa in total parasite proteins extracted from mixed stage cultures in a Western blot (Lane 2) whereas the pre-immune serum does not (Lane 1). To rule out the cross reactivity of α-KAHsp40 antiserum with a highly homologous protein PFE0055c/PF3D7_0501100, we generated antiserum against the same and carried out Western blot analysis. We observed that both α-KAHsp40 and α-PFE antisera gave reactivity with independent bands of different molecular weights without cross reacting with each other (data not shown). All exported proteins undergo processing involving signal peptide cleavage and PEXEL cleavage in the ER before they are targeted to the erythrocyte cytosol. The molecular weight of full length KAHsp40 is 48 kDa which upon processing is reduced to 40 kDa. In order to determine if the *in vivo* form of KAHsp40 is the processed one, we carried out denaturing immunoprecipitation with the metabolically labeled total parasite lysate and the immunoprecipitate (Fig. 1D, lane 2) was run on a 10% SDS-PAGE adjacent to the recombinant His-tagged form of processed KAHsp40 protein (Fig. 1D, lane 1). The purified protein and the *in vivo* form showed similar mobility on SDS-PAGE indicating that the immunoprecipitated form is the processed form. However, the protein shows anomalous mobility as it runs higher than its theoretical molecular weight. The identity of the immunoprecipitated protein was also confirmed by analysis with 2D gel electrophoresis where a single spot of pI 6.3 was obtained (Fig. 1E). Figure S2 shows the protein purification profile of recombinant KAHsp40 along with its MS based validation. Since the affinity purified α-KAHsp40 antibody also pulled down a band of same molecular weight (Fig. S3) from radiolabeled parasite lysate, the rabbit polyclonal antiserum (α-KAHsp40) has been directly used in all the immunological procedures reported here.

### KAHsp40 is Exported to the Erythrocyte Cytosol

**Figure 2:**
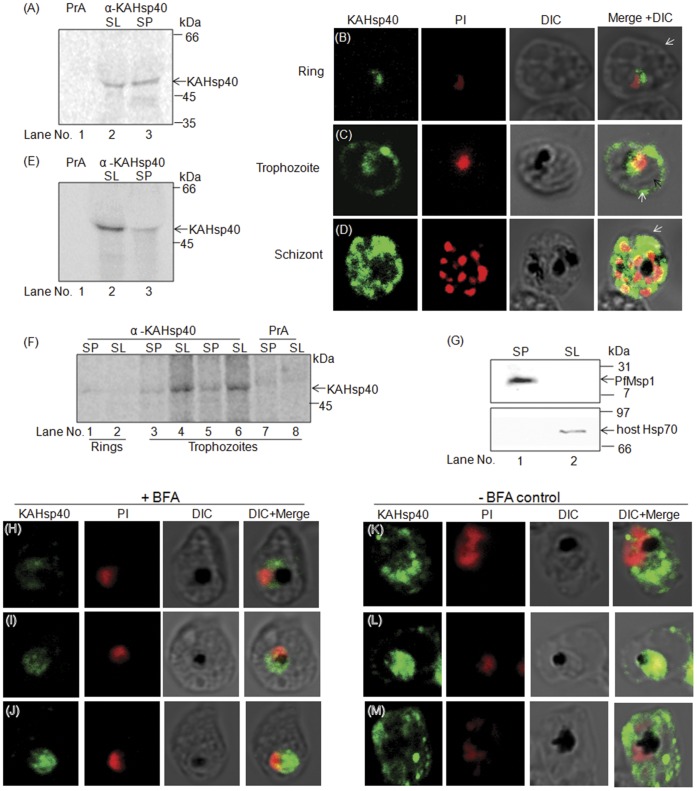
KAHsp40 is exported into the infected RBC cytosol. (A) DIP of fractionated infected RBC with α-KAHsp40 antiserum. SL: Saponin Lysate; SP: Saponin Pellet. This reveals that KAHsp40 is present in both the SP as well as SL. IFA of (B) ring stage parasites (C) trophozoite stage parasites (D) schizont stage parasites reveals the presence of KAHsp40 in discrete foci in the erythrocyte compartment. Black arrow indicates the parasite boundary and white arrow indicates the erythrocyte membrane. (E) Pulse-labeling of the parasite for 15 minutes followed by denaturing IP (DIP) for KAHsp40 reveals that it is exported out to the SL within 15 minutes of synthesis in trophozoite stages. (F) Stage specific denaturing IP of KAHsp40 reveals export of KAHsp40 occurs in trophozoite stages. Lanes 3–4 and 5–6 represent 24 h and 36 h post invasion respectively. (G) Western blot for hHsp70 and PfMsp1 to show that compartment integrity is maintained during saponin based fractionation. (H-J) IFA reveals that the signal for KAHsp40 was restricted to the parasite in BFA treated cultures. (K-M) KAHsp40 was exported to the erythrocyte compartment in mock treated cultures. DIP- Denaturing Immunoprecipitation.

Due to the presence of a PEXEL motif, KAHsp40 is predicted to be exported out of the parasite. To experimentally determine its localization within the infected cell, we carried out denaturing immunoprecipitations (IP) with different fractions of the infected erythrocyte. Infected erythrocytes were first enriched on a 60% percoll gradient from parasite cultures and were subsequently treated with saponin, which allows separation of the erythrocyte cytosol and parasitophorous vacuole (saponin lysate, SL) from the intra-parasitic compartment (saponin pellet, SP). The fractions were denatured and IPs were carried out as described in materials and methods section. As shown in Fig. 2A, denaturing IP revealed the presence of a band corresponding to KAHsp40 in both saponin lysate (SL, Lane 2) and saponin pellet (SP, Lane 3), the infected erythrocyte fraction and the parasite fraction respectively while the protein A control (Lane 1) does not pull down any protein. The results indicated that KAHsp40 is indeed exported out of the parasite, possibly into the erythrocyte cytosol, as predicted by the presence of a PEXEL motif in its N-terminal region.

In order to ascertain the localization of KAHsp40 in the infected erythrocyte, indirect immunofluorescence (IFA) analysis was carried out with acetone fixed asynchronous parasite cultures. KAHsp40 was detected with rabbit α-KAHsp40 followed by FITC-conjugated secondary antibody (Fig. 2B–D, left panel). The parasite nucleus was stained with propidium iodide (PI; Fig. 2B–D, first middle panel). Bright field images were taken (Fig. 2B–D, second middle panel) and overlaid with fluorescent images to examine localization of KAHsp40 within the infected erythrocyte (Fig. 2B–D, right panel). IFA revealed the presence of KAHsp40 in discrete foci in the infected erythrocyte compartment indicative of its presence in either a macromolecular assembly or an organelle. Fig. 2B represents the ring stage of the parasite. It is evident that this Hsp40 was present entirely within the parasite during the ring stage. Fig. 2C and 2D respectively represent the trophozoite and the schizont stages of the parasite. It is evident that KAHsp40 could be detected within the erythrocyte compartment only in the trophozoite stages indicating that its export may be stage specific. In Fig. 2B–D, black arrows indicate parasite boundary and white arrows indicate erythrocyte boundary. This indicates that KAHsp40 is exported out to the infected erythrocyte cytosol beyond the PVM. The pre-immune serum control for IFA is shown in supplemental data (Fig. S4).

To examine stage dependence of KAHsp40 transport, we synchronized parasite cultures and carried out stage specific IPs as well as pulse labeling of the parasites followed by denaturing IPs. For pulse labeling, percoll enriched parasites were pulsed with S^35^ Cys/Met for 15 minutes at trophozoite stages and subjected to saponin lysis followed by denaturing immunoprecipitation of both SP and SL. As evident from Fig. 2E, most of the KAHsp40 was found in the SL (Lane 2) as compared to SP (Lane 3), indicating that KAHsp40 is exported to SL within 15 minutes of its synthesis. Further, stage specific immunoprecipitations (Fig. 2F) indicated that although the KAHsp40 band could be detected at early ring stages (Lanes 1 and 2) where it was found predominantly in the SP, the signal for KAHsp40 could be detected majorly in the SL only at 24 (Lanes 3 and 4) and 36 hours (Lanes 5 and 6) post invasion corresponding to late trophozoite stages. The faint bands observed in SP at 24 and 36 hours represent the newly synthesized pool of KAHsp40 which is yet to be exported. The results suggest that KAHsp40 synthesis begins early following invasion but its export occurs only in the trophozoite stages of the parasite. Further, in trophozoite stages its export is rapid occurring within 15 minutes post synthesis. Western blot analysis for host Hsp70 and PfMsp1 was performed to show that compartment integrity was maintained during saponin based fractionation (Fig. 2G).

Previously, it has been shown that in *P. falciparum*, secretion of many exported proteins is sensitive to Brefeldin-A (BFA) [16], [17]. BFA is a fungal metabolite which causes the re-distribution of the golgi proteins, thus resulting in the block of the classical ER-Golgi secretion pathway [18]. We therefore sought to determine if KAHsp40 trafficking would be BFA sensitive.

BFA treatment was carried out on highly synchronized cultures of *P. falciparum* as described in experimental procedures. In BFA-treated cultures (2H–J), the signal for KAHsp40 can be seen within the parasite only, in close proximity to the nucleus. Whereas, in the mock treated cultures (figure 2K–L), KAHsp40 is trafficked across the confines of the parasite and localizes in different regions of the erythrocyte compartment. Thus, KAHsp40 export is BFA sensitive.

### KAHsp40 does not Associate with Maurer’s Clefts

**Figure 3:**
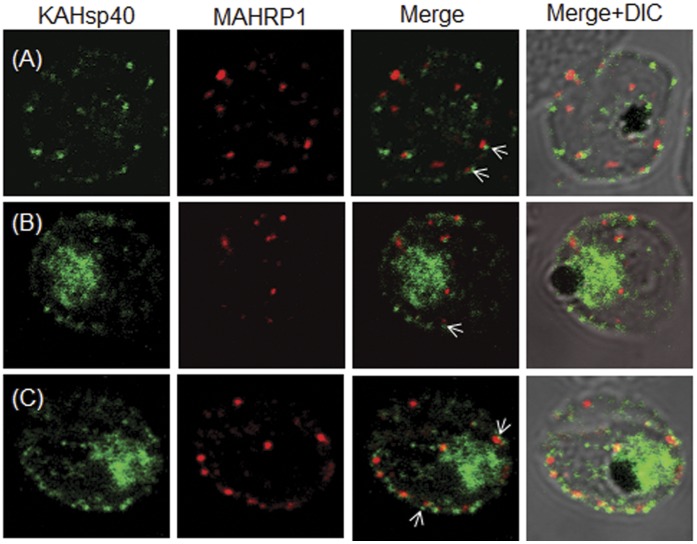
KAHsp40 does not associate with Maurer’s cleft. (A–C) IFA reveals that both KAHsp40 and MAHRP1 are present in discrete foci in the infected erythrocyte, however, they do not co-localize with each other in spite of signals being in close proximity (highlighted by white arrows). The images shown have been taken at the trophozoite stage.

Having established the exported status of KAHsp40, it was important to find out its exact localization in the infected erythrocyte. Previous studies on protein export in malaria parasite have reported number of proteins which associate with Maurer’s clefts, a Golgi-like secretory compartment present in *P. falciparum* infected erythrocyte. These structures have been shown to be important in the trafficking of proteins to RBC membrane. Therefore, we carried out double IFA using antisera against KAHsp40 and MAHRP1, a well characterized Maurer’s cleft marker [19]. KAHsp40 was detected with rabbit α-KAHsp40 followed by FITC-conjugated secondary antibody (Fig. 3A–C, left panel), MAHRP1 was detected with mouse α-MAHRP1 followed by TRITC-conjugated secondary antibody (Fig. 3A–C, middle panel) and the images were overlaid in order to examine co-localization between the two proteins (Fig. 3A–C, right panel). As we can see, the signal for KAHsp40 was found in the parasite as well as the erythrocyte compartment, majorly at the periphery. MAHRP1 formed punctate foci in the erythrocyte cytosol especially underneath the erythrocyte membrane. However, there was no co-localization between the two proteins even though the signals were in close proximity (Fig. 3A–C, merge panel: white arrows).

### KAHsp40 Associates with Knob Components

**Figure 4:**
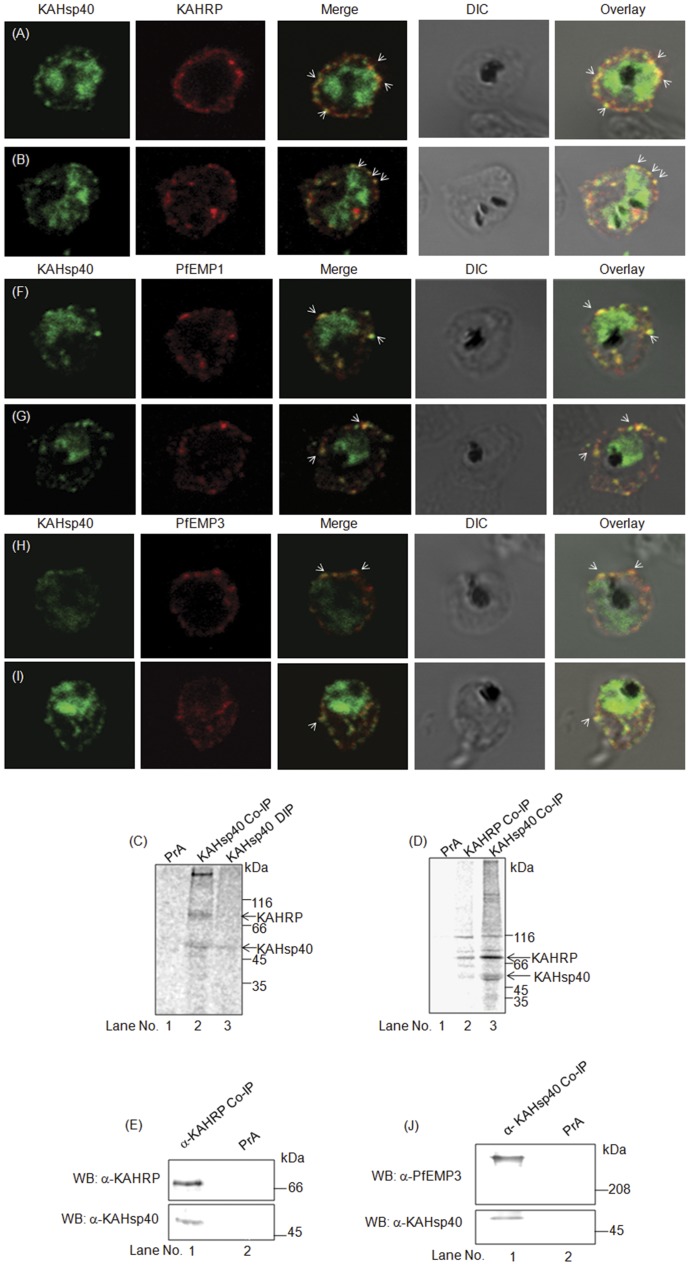
KAHsp40 associates with knobs on the infected erythrocyte membrane. (A-B) IFA reveals that KAHsp40 and KAHRP co-localize in the erythrocyte periphery near the erythrocyte plasma membrane. White arrows indicate the discrete foci in which they co-localize. (C) Co-IP of KAHsp40 from total infected erythrocytes reveals two interactors, of about 66 kDa and 150 kDa. (D) Reciprocal co-IP immunoprecipitates analyzed on a 5–15% gradient gel reveals that 66 kDa interactor of KAHsp40 is KAHRP. (E) Western blot of KAHRP co-IP immunoprecipitate shows the presence of KAHsp40. (F-G) IFA reveals that KAHsp40 and PfEMP1 co-localize with each other on the erythrocyte plasma membrane indicating the close association of KAHsp40 with knobs. (H-I) IFA reveals that KAHsp40 and PfEMP3 co-localize with each other on the erythrocyte plasma membrane indicating the close association of KAHsp40 with knobs. (J) Western blot of KAHsp40 co-IP immunoprecipitate shows the presence of PfEMP3. The images shown have been taken at the trophozoite stage.

Intrigued by the chromosomal juxtaposition of KAHsp40 with KAHRP and PfEMP3, and peripheral localization of KAHsp40 as observed in figure 2 etc., we examined if KAHsp40 also physically associated with these knob proteins. Using KAHRP as a marker for knobs on the infected erythrocyte membrane, we carried out IFA double immunolocalization for KAHsp40 and KAHRP in acetone fixed parasite cultures. KAHsp40 was detected with rabbit α-KAHsp40 followed by FITC-conjugated secondary antibody (Fig. 4A–B, left panel), KAHRP was detected with mouse monoclonal α-KAHRP followed by TRITC-conjugated secondary antibody (Fig. 4A–B, middle panel) and the images were overlaid in order to examine co-localization between the two proteins (Fig. 4A–B, right panel). This revealed partial but distinct co-localization of the two proteins in discrete foci underneath infected erythrocyte plasma membrane in late trophozoite stages of the parasite, as indicated by white arrows in Fig. 4A–B. The results indicate that population of KAHsp40 is associated with KAHRP at any given point of time in mature stages of infected erythrocytes.

In order to examine whether KAHsp40 and KAHRP also interact with each other, we performed co-IPs from non-denatured total infected erythrocyte lysates with α-KAHsp40 antibody, as described under experimental procedures. As shown in Fig. 4C, the α-KAHsp40 antibody co-precipitated two proteins having Mw around 66 kDa and 150 kDa (Lane 2) apart from the KAHsp40 band (identified from denaturing IP in Lane 3). Since KAHsp40 forms a chromosomal cluster and co-localizes with KAHRP in the infected erythrocyte, we looked for KAHRP as a potential interacting partner of KAHsp40. Reciprocal IPs carried out with α-KAHRP and α-KAHsp40 antibodies were run next to each other on a 5–15% gradient gel and mobilities of the co-precipitating bands were compared to each other. As shown in Fig. 3D, α-KAHRP antibody co-precipitated KAHsp40 (Lane 2) and α-KAHsp40 antibody co-precipitated KAHRP (Lane 3) from total infected erythrocyte lysate. The result indicated that KAHsp40 and KAHRP co-localize as well as interact with each other in the infected erythrocyte. Although the co-localization of KAHsp40 with KAHRP is consistently observed, however, there is a partial overlap between the two signals; suggesting a dynamic rather than stable association of KAHsp40 with the membrane-localized KAHRP.

We also performed reciprocal IP to ascertain the association of KAHsp40 with KAHRP. We carried out co-immunoprecipitation on infected erythrocyte lysate using α-KAHRP antibody. The immunoprecipitate was then subjected to immunoblot with α-KAHsp40 antisera and also α-KAHRP antibody (Fig. 4E). A specific signal corresponding to KAHsp40 was obtained thereby validating that the proteins are present in a common complex.

Since the chief function of KAHRP is in knob formation [20], we examined if KAHsp40 had any association with the functional component of knobs, PfEMP1, which is responsible for cytoadherence of the infected erythrocyte. Again, KAHsp40 was detected with rabbit α-KAHsp40 followed by FITC-conjugated secondary antibody (Fig. 4F–G, left panel), PfEMP1 was detected with mouse monoclonal α- PfEMP1 followed by TRITC-conjugated secondary antibody (Fig. 4F–G, middle panel) and the images were overlaid in order to examine co-localization between the two proteins (Fig. 4F–G, right panel). As indicated by white arrows in Fig. 4F–G, double immunolocalization by IFA of KAHsp40 and PfEMP1 revealed that the two proteins co-localized in discrete foci on the periphery of infected erythrocytes.

Having established the association of KAHsp40 with the other components of knob, KAHRP and PfEMP1, we were interested in determining whether KAHsp40 also associates with PfEMP3, the third gene in the chromosomal cluster described above. We carried out IFA to examine the same. KAHsp40 was detected with rabbit α-KAHsp40 followed by FITC-conjugated secondary antibody (Fig. 4H–I, left panel), PfEMP3 was detected with mouse monoclonal α-PfEMP3 followed by TRITC-conjugated secondary antibody (Fig. 4H–I, middle panel) and the images were overlaid in order to examine co-localization between the two proteins (Fig. 4H–I, right panel). As indicated by white arrows in Fig. 4H–I, it can be seen that KAHsp40 and PfEMP3 co-localize partially at discrete foci on the periphery of infected erythrocytes. To determine if PfEMP3 physically associates with KAHsp40, we performed immunoblotting on α-KAHsp40 immunoprecipitates using α-PfEMP3 antibody. A single band corresponding to PfEMP3 was obtained (Fig. 4J) thereby confirming the presence of KAHsp40 in a complex with PfEMP3.

In all, the results indicate close association of KAHsp40 with components of knobs.

### KAHsp40 Associates with the PEXEL Translocon

**Figure 5:**
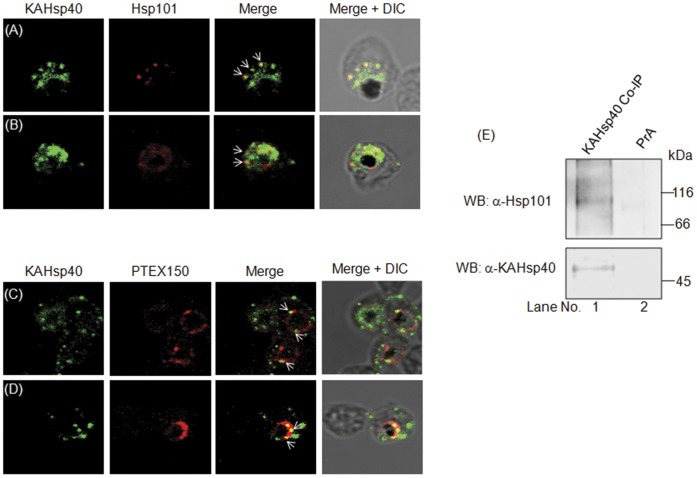
KAHsp40 associates with the PEXEL translocon on the PVM. (A, B) IFA analysis reveals that KAHsp40 and Hsp101 co-localize with each other on the PVM. (C, D) IFA analysis reveals that KAHsp40 and PTEX150 co-localize with each other on the PVM. White arrows indicate the discrete foci in which they co-localize. (E) Western blot of KAHsp40 co-IP immunoprecipitate shows the presence of Hsp101.

Molecular chaperones such as Hsp70 and Hsp40 are known to facilitate protein translocation across membranes. A protein conducting channel known as PEXEL translocon has been recently identified in the PVM [9]. It has been shown to be involved in the trafficking of PEXEL containing proteins to the RBC cytosol. To examine the association of KAHsp40 with the PEXEL translocon, we carried out double immunolocalization by IFA for KAHsp40 with PTEX150 as well as Hsp101, both of which are integral components of the PEXEL translocon. As above, KAHsp40 was detected with rabbit α-KAHsp40 followed by FITC-conjugated secondary antibody (Fig. 5A–D, left panel), Hsp101 or PTEX150 were detected with mouse antisera raised against either Hsp101 or PTEX150 (as described under methods) followed by TRITC-conjugated secondary antibody (Fig. 5A–D, first middle panel) and the images were overlaid in order to examine co-localization between the two proteins (Fig. 5A–D, second middle panel). The merge was overlaid with the bright field image of the parasite (Fig. 5A–D, right panel). Co-localization between proteins is indicated by white arrows in Fig. 5A–D. KAHsp40 was detected in discrete foci in the erythrocyte compartment and also, in addition, around the PVM (Fig. 5A–D, left panel). Hsp101 could be detected around the PVM, in a punctuate pattern (Fig. 5A, B, first middle panel). Co-localization between KAHsp40 and Hsp101 was found in these punctuate structures in the PVM (Fig. 5A, B second middle panel). PTEX150 was found to encircle the parasite (Fig. 5C, D, first middle panel). The discrete foci of KAHsp40 present around the PVM co-localized perfectly with the signal for PTEX150 (Fig. 5C, D, second middle panel). It is clear that KAHsp40 co-localizes with both Hsp101 and PTEX150 in discrete foci in the PVM. To authenticate this association, KAHsp40 co-IP immunoprecipitate was subjected to immunoblot analysis with Hsp101 antibody and a single band corresponding to Hsp101 was obtained (Fig 5E). This further confirmed the association of KAHsp40 with translocon components. However, at this juncture it is unclear whether KAHsp40 is just a substrate of the translocation machinery or a cofactor of the PEXEL translocon having a role to play in translocating the cargo into the erythrocyte.

## Discussion

The study of protein trafficking in the intracellular parasite *Plasmodium falciparum* provides new challenges to cell biologists. By choosing to infect human erythrocytes, *P. falciparum* has denied itself the presence of a well developed compartment system available in other host cells. Yet, trafficking of parasite proteins to the host compartment is critical for parasite survival and pathogenesis of the disease. Recent evidences suggest that unlike pathogens that hitch-hike onto pre-existing host-secretory pathways, the malaria parasite develops its own unique translocation and endomembrane system to deliver essential virulence factors and antigenic proteins to the host cytoplasm and to the surface of the infected erythrocytes. In addition to the classical ER and Golgi compartments, within the parasite, *P. falciparum* also assembles a special translocon at **PVM and membranous compartment called Maurer’s cleft in the ertythrocyte cytoplasm.** One of the most important protein assembly events that occur in the infected erythrocyte is the formation of cytoadherent knobs. Knob formation requires the association of host cytoskeletal components with parasite encoded exported proteins such as PfEMP1 and KAHRP [6]. These are proteins with highly unusual amino acid compositions such as His-repeats in the N-terminal region of KAHRP, highly hydrophobic PfEMP1 containing Glu-homorepeats and a prion-like domain in PfEMP3, which may make these proteins more prone to misfolding and aggregation [8]. Parasite knobs are one of the largest and most complex macromolecular assemblies, measuring 100–120 nm on the erythrocyte plasma membrane [21]. Assembly of such a large and heterogeneous structure may necessitate the involvement of specific chaperones owing to their ability to fold, unfold and stabilize proteins. A recent structural study addressed biophysically the question of how PfEMP1-ATS anchors to host cells in order to facilitate knob assembly [22]. Surprisingly, ATS-KAHRP interaction did not seem to be important for this purpose, however, a PHIST-domain containing parasite protein was found to be involved. It further supports our hypothesis of chaperones being involved in knob assembly as many of the exported chaperones also contain PHIST domains. All chaperone proteins that are predicted to be exported belong to the Hsp40 class. This is not very surprising as Hsp40s have been shown to perform a wide variety of functions in other biological systems and therefore could be the major players for host cell remodeling.

While observing the genomic location of these Hsp40s, we found that PFB0090c/PF3D7_0201800 (KAHsp40) lies along side genes encoding knob proteins KAHRP and PfEMP3, forming a cluster at the subtelomeric end of chromosome 2. It was interesting to note that these genes exhibit an overlap in their time of expression showing transcript peaks within the same time window in the asexual blood stages of the parasite [12]. KAHsp40 turned out to be an exported protein by virtue of a PEXEL signal at its N-terminus. We therefore decided to investigate the localization, export and possible interactors of KAHsp40 in the infected erythrocyte in order to gain better insight into its function.

In agreement to the presence of a PEXEL motif, we found KAHsp40 being exported out to the erythrocyte cytosol in trophozoite stages, which coincides with the appearance of knobs on the infected erythrocyte surface. IFA analysis indicated that KAHsp40 is exported out of the parasite in discrete foci distinct from Maurer’s cleft at the infected erythrocyte periphery. Stage specific IPs revealed that KAHsp40 remains entirely intra-parasitic up to 8 hours post invasion and is exported only in the trophozoite stages. Indeed, we also found KAHsp40 to co-localize with KAHRP on the infected erythrocyte membrane. KAHRP is known to accumulate only on the cytoplasmic side of knobs. KAHsp40 also co-localized partially with PfEMP1 and PfEMP3- the major knob components at the periphery of the infected erythrocyte, indicating its association with knobs. Our co-IP experiments revealed KAHsp40 to be present in a common complex with KAHRP and PfEMP3 further supporting its association with knobs. This indicated that chromosomal cluster of KAHRP, KAHsp40 and PfEMP3 is also a functional cluster. Interaction of KAHsp40 with the components of the PEXEL translocon as well suggests that it may be involved in shuttling proteins (viz. KAHRP, PfEMP3) from PVM to erythrocyte membrane thereby facilitating knob assembly.

The Hsp40 class of chaperones often recruits Hsp70 to facilitate protein folding and assembly events. This recruitment occurs through an HPD motif present within the J domain of the Hsp40 protein [reviewed in 11]. Could it be that the HPD motif present in KAHsp40 recruits an Hsp70 present within the erythrocyte cytoplasm? There are six different Hsp70 isoforms encoded by the parasite. These are 1) PfHsp70-1 (PF3D7_0818900) which is the cytosolic chaperone with EEVD motif, 2) PfHsp70-2 (PF3D7_0917900) or Bip which resides in the ER lumen of the parasite as evident from KDEL motif, 3) PfHsp70-3 (PF3D7_1134000) which is the mitochondrial counterpart, 4) PfHsp70x (PF3D7_0831) which according to the recent annotation, has a signal peptide but ends in EEVN, 5) PfHsp70y (PF3D7_1344200) and 6) PfHsp70z (PF3D7_0708800) [23]. Among these, PfHsp70-x is the one of the least characterized and due to the presence of a signal peptide, it has a potential to be localized in the erythrocyte cytoplasm. In addition to the above parasite encoded Hsp70 isoforms, the host erythrocyte itself has remnant Hsp70 from the erythroid precursors. It is conceivable that exported Hsp40s including KAHsp40 might interact with Hsp70 isoform available in the erythrocyte lumen.

It is important to mention here that knock out of the KAHsp40 gene in CS2 strain of the parasite does not show an obvious defect in knob formation on the erythrocyte surface [24]. This may be due to the presence of several Hsp40s with redundant functions within the erythrocyte cytosol. In addition, knockouts of several molecular chaperones do not result in a direct readable phenotype under normal conditions but often result in phenotypes that can be uncovered only during stressful conditions such as high temperatures or nutrient deprivation or in combination with knockouts of other genes [25]. Although the knockout of KAHsp40 is not essential under normal circumstances, it may be involved in fine-tuning the export and assembly of knob components or recruitment of Hsp70, along with other exported Hsp40.

A recent study has elegantly demonstrated that two exported PfHsp40s (PFE0055c/PF3D7_0501100.1 and PFA0660w/PF3D7_0113700) are present in cholesterol containing mobile structures in the erythrocyte cytosol referred to as J-dots [26]. This study addresses the organization of parasite-encoded Hsp40s in the erythrocyte cytosol for the first time, by examining the fractionation of Hsp40-GFP chimeras into detergent solubilized cells. They found that these Hsp40s are released into the soluble fraction upon treatment of cells with saponin/methyl-β-cyclodextrin, indicating their association with cholesterol-containing structures. The authors have postulated that these structures could be instrumental in protein trafficking within the infected erythrocyte. J-dots are distinct from Maurer’s clefts and do not co-localize with KAHRP and PfEMP1 [26]. Our results reveal non-overlapping distribution of KAHsp40 with J-dots suggesting that these Hsp40s may have different functions. Considering the fact that these Hsp40s cluster together in a phylogram of all 44 PfHsp40s [27], it cannot be ruled out that these Hsp40s could have complementary functions.

In all, our study implicates a parasite encoded Hsp40 that possibly acts in the biogenesis or assembly of cytoadherent knobs. The remodeling of the erythrocyte upon infection by the parasite is crucial for parasite virulence and it is no surprise that the parasite has evolved customized chaperones to facilitate this process. This is also supported by the fact that *Plasmodium vivax* which does not involve host cell remodeling and knob formation in its life cycle lacks the homologs of exported Hsp40s present in *Plasmodium falciparum* [28]. Undoubtedly, implication of additional exported chaperones and understanding the assembly of this fascinating supramolecular complex will be an area of intense research focus in parasitology in the years to come.

## Supporting Information

Figure S1 **Western blot to show the specificity of α-PTEX150 and α-Hsp101 antibodies**. Infected RBC lysate was probed with antisera (Lane No. 1) raised in mice against the C-terminal peptide of PTEX150 and Hsp101. Normal serum was used as control (Lane No. 2). A single band corresponding to the molecular weight of two proteins was obtained indicating the specificity of the raised antisera.
(PDF)

Figure S2 **a). Purification of KAHsp40 protein.** The KAHsp40-pRSET A construct was transformed in *E. Coli.* Rosetta strain and the protein was overexpressed by induction with 0.1 mM IPTG for 4 hrs at 37°C. The protein was purified using Ni-NTA affinity chromatography. The figure shows the coomassie stained profile for protein purification. **b) α**-KAHsp40 specifically recognizes the recombinant form of protein. **c)** MS based identification of the purified protein.
(PDF)

Figure S3 **Immunoprecipitation analysis of KAHsp40**. Metabolically labeled parasite lysate was subjected to IP analysis with affinity purified KAHsp40 antibody and a single specific band was obtained as in the case of western blot & IP with KAHsp40 antisera (Fig. 1C and 2A in the manuscript).
(PDF)

Figure S4 **Immunofluorescence analysis with KAHsp40 pre-immune serum.** No staining was obtained when IFA was done using KAHsp40 pre-immune serum indicating the specificity of α-KAHsp40 antiserum.
(PDF)

## References

[1] Marti M, Good RT, Rug M, Knuepfer E, Cowman AF (2004) Targeting malaria virulence and remodelling proteins to the host erythrocyte. Science 306: 1930–1933. 15591202 10.1126/science.1102452

[2] Hiller NL, Bhattacharjee S, van Ooij C, Liolios K, Harrison T, et al (2004) A host-targeting signal in virulence proteins reveals a secretome in malarial infection. Science 306: 1934–1937. 15591203 10.1126/science.1102737

[3] Lanzer M, Wickert H, Krohne G, Vincensini L, Braun Breton C (2006) Maurer’s clefts: a novel multi-functional organelle in the cytoplasm of *Plasmodium falciparum*- infected erythrocytes. Int J Parasitol 36 23–36. 16337634 10.1016/j.ijpara.2005.10.001

[4] Tilley L, Sougrat R, Lithgow T, Hanssen E (2008) The twists and turns of Maurer’s cleft trafficking in P. falciparum- infected erythrocytes. Traffic 9 187–197. 18088325 10.1111/j.1600-0854.2007.00684.x

[5] Su XZ, Heatwole VM, Wertheimer SP, Guinet F, Herrfeldt JA, et al (1995) The large diverse gene family var encodes proteins involved in cytoadherence and antigenic variation of Plasmodium falciparum-infected erythrocytes. Cell 82 89–100. 7606788 10.1016/0092-8674(95)90055-1

[6] Oh SS, Voigt S, Fisher D, Yi SJ, LeRoy PJ, et al (2000) Plasmodium falciparum erythrocyte membrane protein 1 is anchored to the actin-spectrin junction and knob-associated histidine-rich protein in the erythrocyte skeleton. Mol Biochem Parasitol 108: 237–247. 10838226 10.1016/s0166-6851(00)00227-9

[7] Newbold C, Craig A, Kyes S, Rowe A, Fernandez-Reyes D, et al (1999) Cytoadherence, pathogenesis and the infected red cell surface in Plasmodium falciparum. Int J Parasitol 29: 927–937. 10480730 10.1016/s0020-7519(99)00049-1

[8] Singh GP, Chandra BR, Bhattacharya A, Akhouri RR, Singh SK, et al (2004) Hyper-expansion of asparagines correlates with an abundance of proteins with prion-like domains in Plasmodium falciparum. Mol Biochem Parasitol 137 307–319. 15383301 10.1016/j.molbiopara.2004.05.016

[9] de Koning-Ward TF, Gilson PR, Boddey JA, Rug M, Smith BJ, et al (2009) A newly discovered protein export machine in malaria parasites. Nature 459 945–949. 19536257 10.1038/nature08104 PMC2725363

[10] Gehde N, Hinrichs C, Montilla I, Charpian S, Lingelbach K, et al (2009) Protein unfolding is an essential requirement for transport across the parasitophorous vacuolar membrane of Plasmodium falciparum. Mol Microbiol 71 (3): 613–628. 10.1111/j.1365-2958.2008.06552.x 19040635

[11] Walsh P, Bursac D, Law YC, Cyr D, Lithgow T (2004) The J-protein family: modulating protein assembly, disassembly and translocation. EMBO Rep 5 567–571. 15170475 10.1038/sj.embor.7400172 PMC1299080

[12] Biggs BA, Kemp DJ, Brown GV (1989) Subtelomeric chromosome deletions in field isolates of Plasmodium falciparum and their relationship to loss of cytoadherence in vitro. Proc Natl Acad Sci U S A 86 2428–2432. 2648403 10.1073/pnas.86.7.2428 PMC286926

[13] Trager W, Jensen JB (1976) Human malaria parasites in continuous culture. Science 193: 673–5. 781840 10.1126/science.781840

[14] Rug M, Prescott SW, Fernandez KM, Cooke BM, Cowman AF (2006) The role of KAHRP domains in knob formation and cytoadherence of *P. falciparum*- infected human erythrocytes. Blood 108 370–378. 16507777 10.1182/blood-2005-11-4624 PMC1895844

[15] Knuepfer E, Rug M, Klonis N, Tilley L, Cowman AF (2005) Trafficking determinants for PfEMP3 export and assembly under the Plasmodium falciparum-infected red blood cell membrane. MolMicrobiol 58 1039–1053. 10.1111/j.1365-2958.2005.04895.x 16262789

[16] Khattab A, Klinkert MQ (2005) Maurer’s clefts-restricted localization, orientation and export of a *Plasmodium falciparum* RIFIN. Traffic 7: 1654–1665. 10.1111/j.1600-0854.2006.00494.x 17014697

[17] Saridaki T, Sanchez CP, Pfahler J, Lanzer M (2008) A conditional export system provides new insights into protein export in Plasmodium falciparum-infected erythrocytes. Cell Microbiol 10(12): 2483–2495. 18691247 10.1111/j.1462-5822.2008.01223.x

[18] Lippincott-Schwartz J, Yuan LC, Bonifacino JS, Klausner RD (1989) Rapid redistribution of Golgi proteins into the ER in cells treated with brefeldin A: evidence for membrane cycling from Golgi to ER. Cell 56: 801–813. 2647301 10.1016/0092-8674(89)90685-5 PMC7173269

[19] Spycher C, Spycher C, Rug M, Klonis N, Ferguson DJ, et al (2006) Genesis of and trafficking to the Maurer’s clefts of Plasmodium falciparum-infected erythrocytes. Mol Cell Biol 26(11): 4074–85. 16705161 10.1128/MCB.00095-06 PMC1489082

[20] Crabb BS, Cooke BM, Reeder JC, Waller RF, Caruana SR, et al (1997) Targeted gene disruption shows that knobs enable malaria-infected red cells to cytoadhere under physiological shear stress. Cell 1997 89(2): 287–96. 10.1016/s0092-8674(00)80207-x 9108483

[21] Aikawa M, Kamanura K, Shiraishi S, Matsumoto Y, Arwati H, et al (1996) Membrane knobs of unfixed Plasmodium falciparum infected erythrocytes: new findings as revealed by atomic force microscopy and surface potential spectroscopy. Exp Parasitol 84 339–343. 8948323 10.1006/expr.1996.0122

[22] Mayer C, Slater L, Erat MC, Konrat R, Vakonakis I (2012) Structural analysis of the Plasmodium falciparum erythrocyte membrane protein 1 (PfEMP1) intracellular domain reveals a conserved interaction epitope. J Biol Chem 287(10): 7182–9. 22249178 10.1074/jbc.M111.330779 PMC3293552

[23] Shonhai A, Boshoff A, Blatch GL (2007) The structural and functional diversity of Hsp70 proteins from *Plasmodium falciparum.* . Protein Sci 16: 1803–1818. 17766381 10.1110/ps.072918107 PMC2206976

[24] Maier AG, Rug M, O’Neill MT, Brown M, Chakravorty S, et al (2008) Exported proteins required for virulence and rigidity of Plasmodium falciparum-infected human erythrocytes. Cell 134 48–61. 18614010 10.1016/j.cell.2008.04.051 PMC2568870

[25] Genevaux P, Georgopoulos C, Kelley WL (2007) The Hsp70 chaperone machines of Escherichia coli: a paradigm for the repartition of chaperone functions. MolMicrobiol 66 840–857. 10.1111/j.1365-2958.2007.05961.x 17919282

[26] Kulzer S, Rug M, Brinkmann K, Ping C, Cowman A (2010) et.al (2010) Parasite-encoded Hsp40 proteins define novel mobile structures in the cytosol of the *P. falciparum*- infected erythrocyte. Cell Microbiol 12(10): 1398–420. 20482550 10.1111/j.1462-5822.2010.01477.x

[27] Acharya P, Kumar R, Tatu U (2007) Chaperoning a cellular upheaval in malaria: heat shock proteins in Plasmodium falciparum. Mol Biochem Parasitol 153: 85–94. 17307260 10.1016/j.molbiopara.2007.01.009

[28] Botha M, Pesce ER, Blatch GL (2007) The Hsp40 proteins of Plasmodium falciparum and other apicomplexa: regulating chaperone power in the parasite and the host. Int J Biochem Cell Biol 39 1783–1803. 10.1016/j.biocel.2007.02.011 17428722

