# Supplementary material for: An Exported Heat Shock Protein 40 Associates with Pathogenesis-Related Knobs in Plasmodium falciparum Infected Erythrocytes
Source: PLoS One. 2012 Sep 7;7(9):e44605. doi: 10.1371/journal.pone.0044605 (PMC3436795; doi:10.1371/journal.pone.0044605)
Supplement: Figure S1 — Western blot to show the specificity of α-PTEX150 and α-Hsp101 antibodies. Infected RBC lysate was probed with antisera (Lane No. 1) raised in mice against the C-terminal peptide of PTEX150 and Hsp101. Normal serum was used as control (Lane No. 2). A single band corresponding to the molecular weight of two proteins was obtained indicating the specificity of the raised antisera. (PDF) [file pone.0044605.s001.pdf]

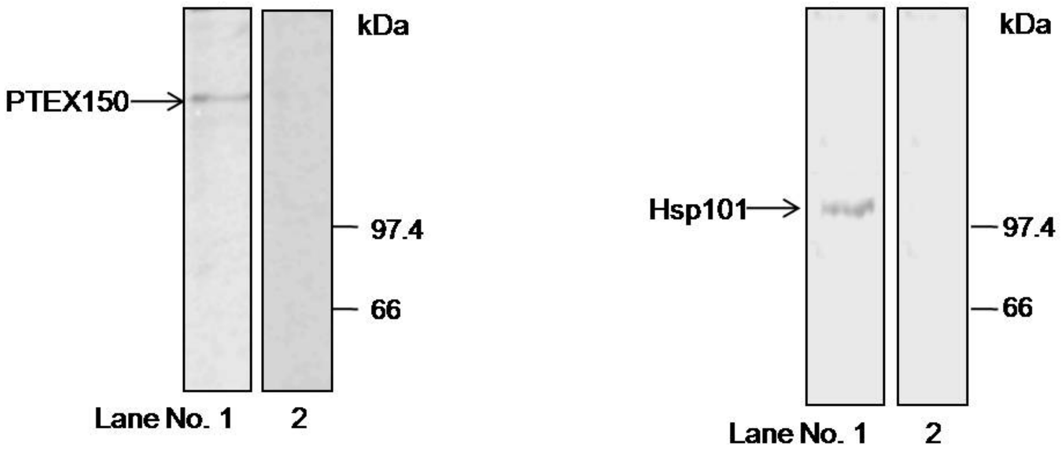

Figure S1: Western blot to show the specificity of α-PTEX150 and α-Hsp101 antibodies. Infected RBC lysate was probed with antisera (Lane No. 1) raised in mice against the C-terminal peptide of PTEX150 and Hsp101. Normal serum was used as control (Lane No. 2). A single band corresponding to the molecular weight of two proteins was obtained indicating the specificity of the raised antisera.
