# Supplementary material for: An Exported Heat Shock Protein 40 Associates with Pathogenesis-Related Knobs in Plasmodium falciparum Infected Erythrocytes
Source: PLoS One. 2012 Sep 7;7(9):e44605. doi: 10.1371/journal.pone.0044605 (PMC3436795; doi:10.1371/journal.pone.0044605)
Supplement: Figure S2 — a). Purification of KAHsp40 protein. The KAHsp40-pRSET A construct was transformed in E. Coli. Rosetta strain and the protein was overexpressed by induction with 0.1 mM IPTG for 4 hrs at 37°C. The protein was purified using Ni-NTA affinity chromatography. The figure shows the coomassie stained profile for protein purification. b) α-KAHsp40 specifically recognizes the recombinant form of protein. c) MS based identification of the purified protein. (PDF) [file pone.0044605.s002.pdf]

a)

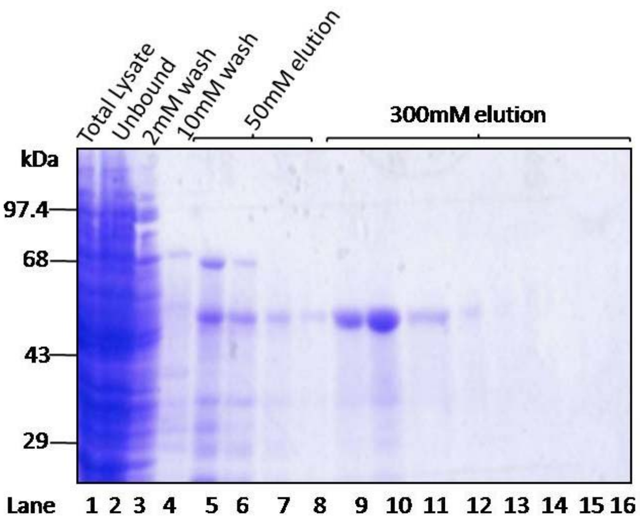
Total Lysate
Unbound
2mM wash
10mM wash
50mM elution
300mM elution
kDa
97.4
68
43
29
Lane 1 2 3 4 5 6 7 8 9 10 11 12 13 14 15 16

b)

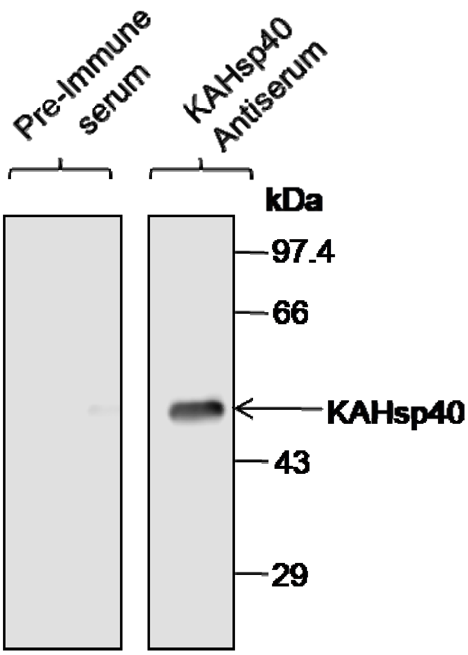
Pre-Immune serum
KAHsp40 Antiserum
kDa
97.4
66
KAHsp40
43
29

c)

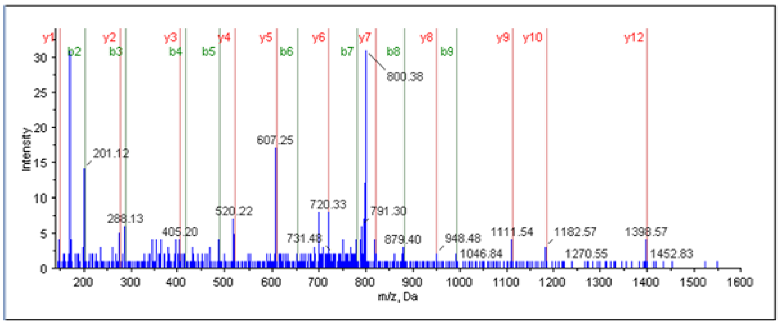

Proteins Detected

N	Unused	Total	% Cov	Accession #	Name	Species	Peptides(95%)	Biological Processes	Molecular Fu
1	36.76	36.76	53.4	trm|O96123	Hypothetical protein PFB0090c	Plasmodium falciparum	33	Protein metabolism ...	Chaperone->

Protein Sequence Coverage - Hypothetical protein PFB0090c

MKDKYRIDCNIYIYILIEKFQDQIQTSYYNKNNTSGNVSNLIIKRNLAQTQRNFKSKNGKASTKKNEDYYSILGVSRDCTNEDIKKAYKKLAMKWHPDKHLNAASKKEADNMFKSIS
EAYEVLSDEEKRDIYDKYGEEGLDKYGSNNGHSKGFKRTDPNDVFSKFFKTETKFYSNSPSSPNGNVLFEGSLFGGSSPFSGINPRSGSGYTTSKSFSSMDKVEEYVVPLYVTLEDL
YNGTQKKLKVTRKRCQGVTTYDDEFFVTVDIKSGWCDGTTITYKGEGDQTSPMSNPGDLVFTIKTVDHDRFVRSYNDLIYRCPITLEQALTGHKFTIITLDNRDIDIQVDEIVTPLT
TRVITSEGMPYMENPKMKGNLIIEFDIIFPKKLSDEQKELIKEALGGNGF

Figure S2 a): Purification of KAHsp40 protein. The KAHsp40-pRSET A construct was transformed in *E. Coli.* Rosetta strain and the protein was overexpressed by induction with 0.1mM IPTG for 4hrs at 37°C. The protein was purified using Ni-NTA affinity chromatography. The figure shows the coomassie stained profile for protein purification. **b)** α-KAHsp40 specifically recognizes the recombinant form of protein. **c)** MS based identification of the purified protein.
