# Supplementary material for: An Exported Heat Shock Protein 40 Associates with Pathogenesis-Related Knobs in Plasmodium falciparum Infected Erythrocytes
Source: PLoS One. 2012 Sep 7;7(9):e44605. doi: 10.1371/journal.pone.0044605 (PMC3436795; doi:10.1371/journal.pone.0044605)
Supplement: Figure S3 — Immunoprecipitation analysis of KAHsp40. Metabolically labeled parasite lysate was subjected to IP analysis with affinity purified KAHsp40 antibody and a single specific band was obtained as in the case of western blot & IP with KAHsp40 antisera (Fig. 1C and 2A in the manuscript). (PDF) [file pone.0044605.s003.pdf]

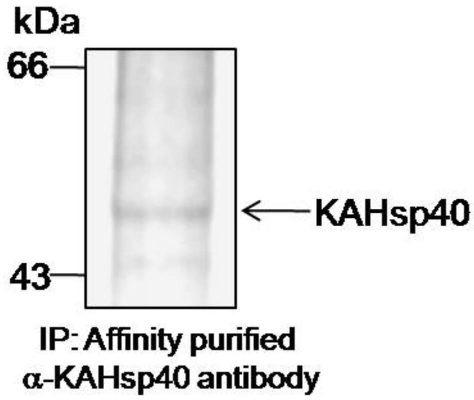

Figure S3: Immunoprecipitation analysis of KAHsp40. Metabolically labeled parasite lysate was subjected to IP analysis with affinity purified KAHsp40 antibody and a single specific band was obtained as in the case of western blot & IP with KAHsp40 antisera (Fig. 1C and 2A in the manuscript).
