# Supplementary material for: An Exported Heat Shock Protein 40 Associates with Pathogenesis-Related Knobs in Plasmodium falciparum Infected Erythrocytes
Source: PLoS One. 2012 Sep 7;7(9):e44605. doi: 10.1371/journal.pone.0044605 (PMC3436795; doi:10.1371/journal.pone.0044605)
Supplement: Figure S4 — Immunofluorescence analysis with KAHsp40 pre-immune serum. No staining was obtained when IFA was done using KAHsp40 pre-immune serum indicating the specificity of α-KAHsp40 antiserum. (PDF) [file pone.0044605.s004.pdf]

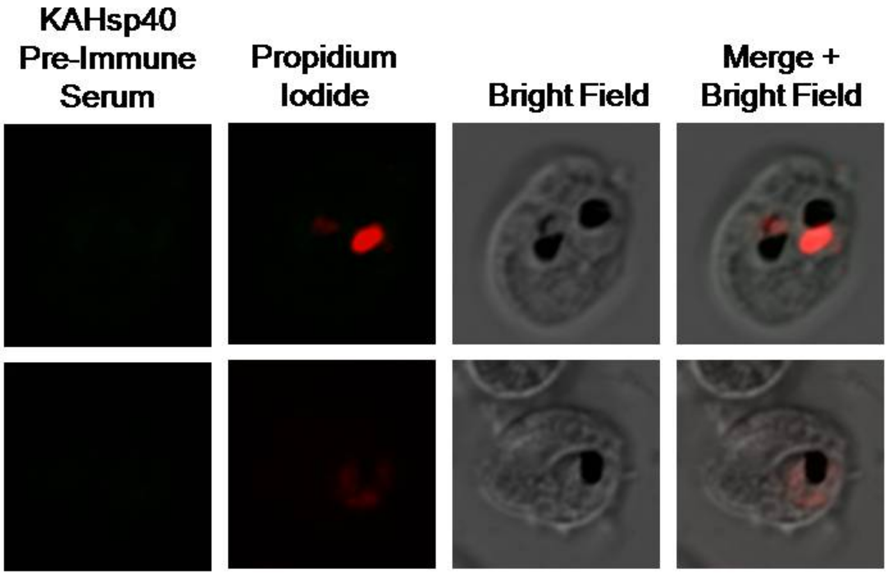

Figure S4: Immunofluorescence analysis with KAHsp40 pre-immune serum. No staining was obtained when IFA was done using KAHsp40 pre-immune serum indicating the specificity of α-KAHsp40 antiserum.
